# Natural Language Processing for Assessing Quality Indicators in Free-Text Colonoscopy and Pathology Reports: Development and Usability Study

**DOI:** 10.2196/35257

**Published:** 2022-04-15

**Authors:** Jung Ho Bae, Hyun Wook Han, Sun Young Yang, Gyuseon Song, Soonok Sa, Goh Eun Chung, Ji Yeon Seo, Eun Hyo Jin, Heecheon Kim, DongUk An

**Affiliations:** 1 Department of Biomedical Informatics CHA University School of Medicine CHA University Seongnam Republic of Korea; 2 Institute for Biomedical Informatics CHA University School of Medicine CHA University Seongnam Republic of Korea; 3 Department of Internal Medicine and Healthcare Research Institute Healthcare System Gangnam Center Seoul National University Hospital Seoul Republic of Korea; 4 Miso Info Tech Co, Ltd Seoul Republic of Korea

**Keywords:** natural language processing, colonoscopy, adenoma, endoscopy

## Abstract

**Background:**

Manual data extraction of colonoscopy quality indicators is time and labor intensive. Natural language processing (NLP), a computer-based linguistics technique, can automate the extraction of important clinical information, such as adverse events, from unstructured free-text reports. NLP information extraction can facilitate the optimization of clinical work by helping to improve quality control and patient management.

**Objective:**

We developed an NLP pipeline to analyze free-text colonoscopy and pathology reports and evaluated its ability to automatically assess adenoma detection rate (ADR), sessile serrated lesion detection rate (SDR), and postcolonoscopy surveillance intervals.

**Methods:**

The NLP tool for extracting colonoscopy quality indicators was developed using a data set of 2000 screening colonoscopy reports from a single health care system, with an associated 1425 pathology reports. The NLP system was then tested on a data set of 1000 colonoscopy reports and its performance was compared with that of 5 human annotators. Additionally, data from 54,562 colonoscopies performed between 2010 and 2019 were analyzed using the NLP pipeline.

**Results:**

The NLP pipeline achieved an overall accuracy of 0.99-1.00 for identifying polyp subtypes, 0.99-1.00 for identifying the anatomical location of polyps, and 0.98 for counting the number of neoplastic polyps. The NLP pipeline achieved performance similar to clinical experts for assessing ADR, SDR, and surveillance intervals. NLP analysis of a 10-year colonoscopy data set identified great individual variance in colonoscopy quality indicators among 25 endoscopists.

**Conclusions:**

The NLP pipeline could accurately extract information from colonoscopy and pathology reports and demonstrated clinical efficacy for assessing ADR, SDR, and surveillance intervals in these reports. Implementation of the system enabled automated analysis and feedback on quality indicators, which could motivate endoscopists to improve the quality of their performance and improve clinical decision-making in colorectal cancer screening programs.

## Introduction

High-quality colonoscopy is a proven method of reducing colorectal cancer risk by allowing early detection and removal of premalignant polyps [1]. However, there are considerable variations in the quality of colonoscopies performed by endoscopists [2-4]. Therefore, quality assurance is an essential part of colonoscopy screening programs, and the American Society of Gastrointestinal Endoscopy/American College of Gastroenterology Task Force on Quality in Endoscopy has published indicators for colonoscopy to improve safety and quality [5]. While all the indicators are important, the adenoma detection rate (ADR) and sessile serrated lesion (SSL) detection rate (SDR) of endoscopists are well-established key indicators of postcolonoscopy colorectal cancer incidence and related deaths [5-7]. Another crucial quality indicator is the adherence to guidelines for setting the frequency of follow-up colonoscopies, known as the surveillance interval. Recommending an incorrect surveillance interval may increase the incidence of metachronous lesion or lead to the overuse of colonoscopies [8].

Periodically reporting to endoscopists their performance on quality measures effectively improves the quality of colonoscopies by encouraging introspection and motivation for behavior changes [9-11]. However, reporting ADR, SDR, and surveillance intervals requires careful manual review of colonoscopy reports and their associated pathology reports and following this review with a calculation of polyp data based on clinical guidelines. This series of processes for quality reporting is laborious and time-consuming.

Natural language processing (NLP) is a computer-based linguistics technique used to extract information from free-text data documents [12]. NLP allows the automation of report creation by extracting important clinical information from unstructured free-text documents. NLP has been used in various clinical fields [12-17]. The application of NLP to information extraction requires identifying clinical information, such as adverse events, and facilitates various aspects of optimizing clinical work, such as quality control and patient management [18].

Here, we developed an NLP pipeline for the automated assessment of quality indicators, such as ADR, SDR, and surveillance intervals, from multi-language colonoscopy and pathology report forms. The pipeline was evaluated in a validation set and compared with expert manual reviews to determine whether the pipeline could reliably assist the inefficient manual process. The NLP system was also applied to a 10-year set of colonoscopy and pathology reports to investigate its ability to process real-world data on colonoscopy quality indicators from individual endoscopists.

## Methods

### Study Design and Population

**Figure 1:**
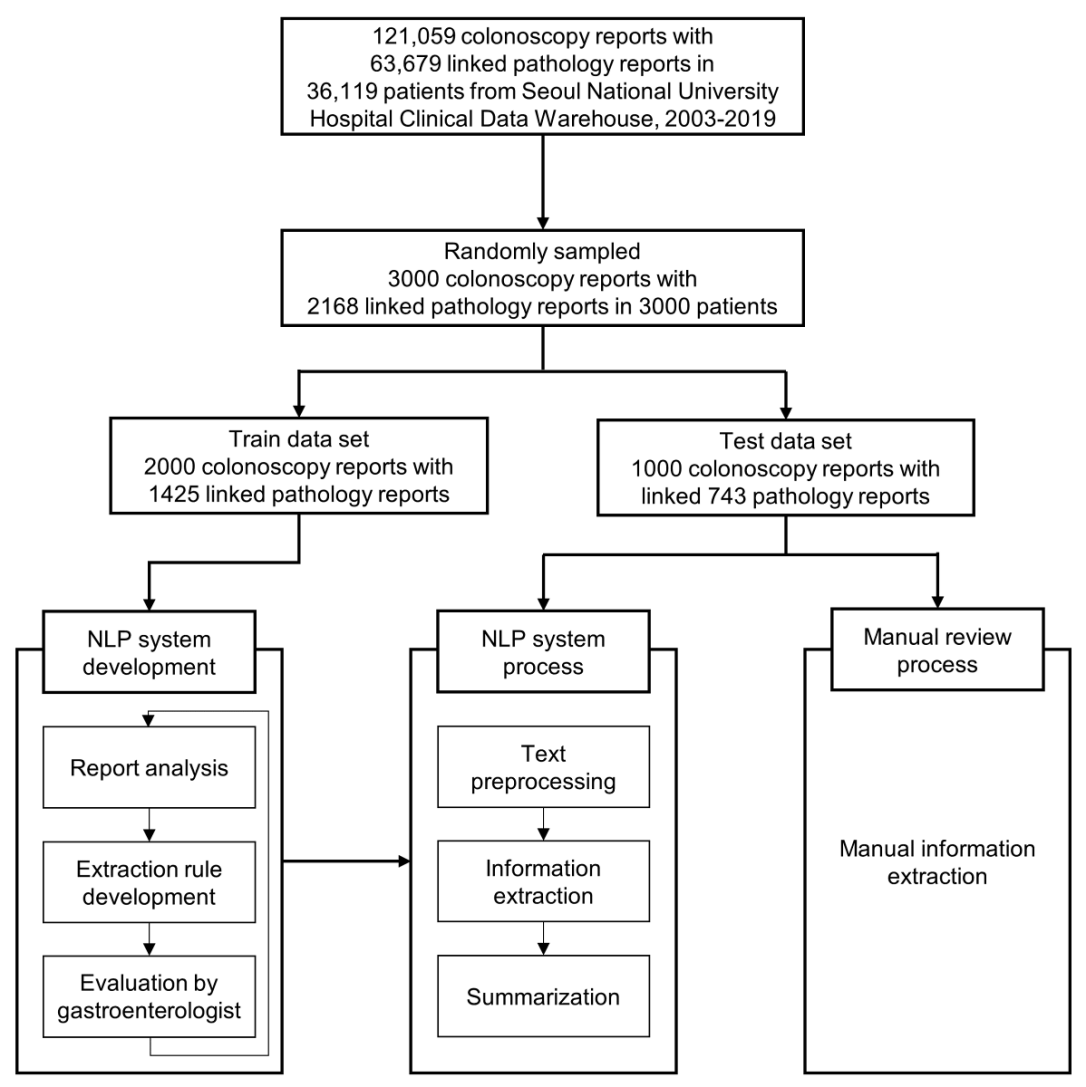
Data set description and process for the NLP pipeline development and information extraction. NLP: natural language processing.

Colonoscopy for colon cancer screening was performed at Seoul National University Hospital Gangnam Center, where comprehensive medical checkups of approximately 30,000 patients are conducted annually. A total of 121,059 screening and surveillance colonoscopies with 63,697 associated pathology reports from 36,119 patients examined between 2003 and 2019 were derived from SUPREME (Seoul National University Hospital Patients Research Environment), the clinical data warehouse of Seoul National University Hospital. A representative sample of 3000 colonoscopy reports, paired with 2168 pathology reports, from 3000 patients examined after 2003 was randomly selected and used as the development data set for the NLP pipeline (Figure 1). The reports were divided into a training data set of 2000 colonoscopy reports for NLP rule formulation and a testing data set of 1000 colonoscopy reports for validation. Five human annotators (4 board-certified gastroenterologists and 1 researcher) manually reviewed all procedure data and made reference to a consensus of the 5 human annotators for the data set.

### NLP Pipeline Development

We used regular expressions in Python (3.7.10, Python Software Foundation) and smartTA (1.0b, MISO Info Tech) to develop the NLP pipeline. Regular expressions are a sequence of characters specialized for complex text processing using metacharacters [19]. smartTA is NLP software that helps analyze linguistic patterns and construct lexicons. The NLP pipeline was developed with the following steps: First, we developed multi-language report forms (in Korean only, in English only, and a mixed report form) for the NLP pipeline processing by creating a Korean-English lexicon for medical terms, synonyms, and endoscopic abbreviations using a training data set and a colonoscopy textbook [20]. Second, we determined removable terms and phrases in the reports through an interactive discussion with gastroenterologists. Third, we defined the extraction rules using smartTA. Fourth, we updated the rules after the extracted results were evaluated by gastroenterologists. These development steps were repeated until it was no longer possible to obtain performance increases by updating the extraction rules. The final version was validated using the 1000-report testing data set.

**Figure 2:**
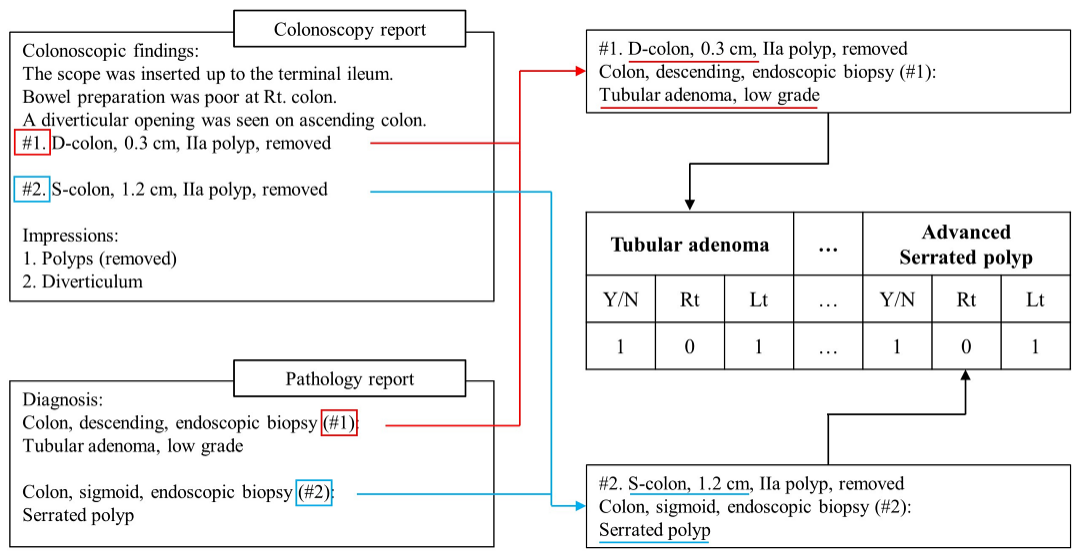
Extraction and summarization process of the NLP pipeline. NLP: natural language process; Y/N: yes/no (indicating presence or absence); Rt: right colon; Lt: left colon.

The NLP pipeline developed for this study consisted of text preprocessing, information extraction, and summarization (Figure 1, Figure 2). In text preprocessing, the colonoscopy and associated pathology reports were combined as follows: each sentence including a biopsy-related phrase (ie, an abbreviation, number, or character) in the findings section of the colonoscopy report was linked with polyp histopathology results in the diagnosis section of the pathology report according to the sequence of specimens in the pathology report. In information extraction, the pipeline consulted the lexicon to extract the target information, including the presence, type, location, and size of polyps, from the combined colonoscopy-pathology text.

Finally, the extracted information on the biopsied polyps was summarized in the final summary format and used to calculate the detection rate and surveillance interval.

### Target Variables for Polyp Detection and Surveillance Interval Measurement

The NLP tool extracted specific information on colon polyps, such as pathological type, anatomical location, and size. The type of colon polyp was extracted from the pathology reports and categorized as adenoma, serrated polyp, or carcinoma. Additionally, the NLP tool extracted the subcategory for adenomas (ie, tubular, tubulovillous, villous, or adenoma with high-grade dysplasia) and serrated polyps (ie, hyperplastic polyp, SSL, or traditional serrated adenoma). Information on the anatomical location of polyps was extracted from the findings section of the colonoscopy reports and defined as follows: left-colon polyps were defined as those located between the rectum and the splenic flexure (ie, the rectum, rectosigmoid, sigmoid, descending colon, and splenic flexure); right-colon polyps were defined as those located between the transverse colon and the cecum (ie, the transverse colon, hepatic flexure, ascending colon, cecum, and ileocecal valve). When location measurements were provided as the distance from the anal verge in cm, a distance of ≥60 cm was considered to be in the right colon.

The detection rate was calculated as the proportion of colonoscopies that detected at least 1 adenoma or SSL; the overall detection rate and the per-physician detection rate were calculated. The detection rate for advanced adenoma was defined as the proportion of screening colonoscopies that detected a polyp with size ≥1 cm or an adenomatous pathology with high-grade dysplasia or villous features. The detection rate for advanced SSL was defined as the proportion of screening colonoscopies that detected a polyp with a size ≥1 cm or a pathology with low- or high-grade dysplasia. Surveillance intervals were chosen based on the 2020 US Multi-Society Task Force guidelines, which recommend that a patient with neoplastic polyps undergo surveillance colonoscopies at 1 of 6 defined intervals [21].

### Statistical Analysis and Performance Evaluation

Continuous variables were calculated as the mean (SD). Discrete data were tabulated as numbers and percentages. The chi-square test was used to compare proportions, and a 2-tailed *t* test was used to compare quantitative variables. Information extraction performance was evaluated by recall, precision, accuracy, and the F1 score. The F1 score is the harmonic mean of precision and recall. Python (3.7.10) and the SciPy package (1.6.2) were used for statistical calculations [22].

### Analysis of a 10-Year Set of Colonoscopy Reports for ADR, SDR, and Surveillance Interval

The NLP pipeline analyzed 54,562 screening and surveillance colonoscopy reports and 34,943 associated pathology reports from 12,264 patients aged ≥50 years at Seoul National University Hospital Gangnam Center; all patients were examined between January 2010 and December 2019. The ADR, SDR, and surveillance intervals were investigated, both overall and individually for endoscopists who performed >500 procedures. The relationship between the polyp detection rate and surveillance interval was also determined.

### Ethics Approval

This study was approved by the Institutional Review Board of Seoul National University Hospital (1909-093-670).

## Results

### NLP Information Extraction Performance

Table 1 shows the demographics of the 2000-report training data set and the 1000-report testing data set for the NLP pipeline. The NLP tool extracted variables to calculate the quality indicators. Table 2 shows the extracted key information on pathological type, including advanced features, location, and the number of polyps, which was assessed for recall, precision, accuracy, and the F1 score in the testing data set. The performance of the NLP pipeline ranged from 0.97 to 1.00 in all performance metrics for the presence of adenomas and SSLs with advanced features. For the location of colon polyps, the NLP pipeline demonstrated excellent performance for adenomas, ranging from 0.97 to 1.00; however, the NLP pipeline demonstrated a relatively lower performance for detecting SSL location. The NLP pipeline also demonstrated high performance (>0.98) for counting the number of adenomas and SSLs.

**Table 1:** Characteristics of training and testing data sets for the development of the natural language processing pipeline.

Characteristics		Training (N=2000)	Testing (N=1000)	*P* value	
Age, mean (SD)		58.6 (6.4)	60.4 (6.5)	<.001	
**Sex**					.86
	Male, n (%)	1188 (59.4)	590 (59.0)		
	Female, n (%)	812 (40.6)	410 (41.0)		
**Adenoma**					
	Overall, n (%)	925 (46.2)	475 (47.5)	.72	
	Right colon only, n (%)	501 (25.0)	265 (26.5)	.54	
	Left colon only, n (%)	212 (10.6)	113 (11.3)	.65	
	Both, n (%)	212 (10.6)	97 (9.7)	.53	
**Advanced adenoma^a^**					
	Overall, n (%)	77 (3.8)	34 (3.4)	.62	
	Right colon only, n (%)	51 (2.6)	14 (1.4)	.06	
	Left colon only, n (%)	24 (1.2)	18 (1.8)	.26	
	Both, n (%)	3 (0.2)	2 (0.2)	.87	
**Sessile serrated lesion**					
	Overall, n (%)	121 (6)	66 (6.6)	.64	
	Right colon only, n (%)	79 (4)	45 (4.5)	.56	
	Left colon only, n (%)	34 (1.7)	15 (1.5)	.80	
	Both, n (%)	8 (0.4)	6 (0.6)	.64	
**Advanced sessile serrated lesion^b^**					
	Overall, n (%)	19 (1)	12 (1.2)	.66	
	Right colon only, n (%)	14 (0.7)	10 (1)	.52	
	Left colon only, n (%)	4 (0.2)	1 (0.1)	.88	
	Both, n (%)	1 (0.1)	1 (0.1)	.80	
**Cancer**					
	Overall, n (%)	3 (0.2)	0 (0)	.54	
	Right colon only, n (%)	0 (0)	0 (0)		
	Left colon only, n (%)	3 (0.2)	0 (0)	.54	
	Both, n (%)	0 (0)	0 (0)		

^a^Advanced adenomas were defined as adenomas ≥1 cm in size or with pathological features such as high-grade dysplasia or villous features.

^b^Advanced sessile serrated lesions were defined as lesions ≥1 cm in size or with pathological features such as low or high-grade dysplasia.

**Table 2:** Performance of the natural language processing pipeline in the testing data set (N=1000).

Indicators			Recall		Precision		Accuracy	F1 score
Presence of a conventional adenoma			0.99		1.00		0.99	0.99
**Location of conventional adenoma**								
	None	1.00		0.98		0.99		0.99
	Right colon only	0.98		1.00		0.99		0.99
	Left colon only	0.98		0.99		0.99		0.99
	Both	0.99		0.97		0.99		0.98
Presence of an advanced adenoma^a^			1.00		0.97		0.99	0.99
**Location of advanced adenoma**								
	None	0.99		1.00		0.99		0.99
	Right colon only	1.00		0.93		0.99		0.97
	Left colon only	1.00		1.00		1.00		1.00
	Both	1.00		1.00		1.00		1.00
Presence of an SSL^b^			0.98		1.00		0.99	0.99
**Location of SSL**								
	None	1.00		0.99		0.99		0.99
	Right colon only	0.96		1.00		0.99		0.98
	Left colon only	1.00		1.00		1.00		1.00
	Both	1.00		0.86		0.99		0.92
Presence of an advanced SSL^c^			1.00		1.00		1.00	1.00
**Location of advanced SSL**								
	None	1.00		1.00		1.00		1.00
	Right colon only	0.90		1.00		0.99		0.95
	Left colon only	1.00		1.00		1.00		1.00
	Both	1.00		0.50		0.99		0.67
**Total number of adenomas**								
	0	1.00		0.99		1.00		0.99
	1-2	0.99		0.99		0.99		0.99
	3-4	0.98		1.00		0.98		0.99
	5-10	1.00		1.00		1.00		1.00
	>10	N/A^d^		N/A		N/A		N/A
**Total number of SSLs**								
	0	1.00		0.99		1.00		0.99
	1-2	0.98		1.00		0.98		0.99
	3-4	1.00		1.00		1.00		1.00
	5-10	N/A		N/A		N/A		N/A

^a^Advanced adenomas were defined as adenomas ≥1 cm in size or with pathological features such as high-grade dysplasia or villous features.

^b^SSL: sessile serrated lesion.

^c^Advanced sessile serrated lesions were defined as lesions ≥1 cm in size or with pathological features such as low or high-grade dysplasia.

^d^N/A: not applicable.

### NLP Performance in Calculating Colonoscopy Quality Indicators

The NLP pipeline assessed the mean ADR and SDR in the test data set as 47.2% (472/1000) and 6.5% (65/1000), respectively. The gold standard evaluation assessed these values as 47.5% (475/1000) and 6.6% (66/1000), respectively (Table 3). The differences in assessed ADR and SDR between the manual review, the NLP pipeline, and the gold standard values were not significant. For assessing the number of patients assigned to each of the 6 surveillance interval groups described in the 2020 US Multi-Society Task Force guidelines, the NLP pipeline and manual review demonstrated similar performance; however, the NLP pipeline demonstrated a relatively higher accuracy in assessing the number of patients assigned to the 3-year group than the manual review (63/63, 100% vs 59/63, 93.6%, respectively); this was also true for the 3-5-year group (68/69, 98.6% vs 65/69, 94.2%, respectively). It is a complicated task to assess risk stratification in these groups.

**Table 3:** Comparison of polyp detection rate and surveillance interval group assignment as assessed by manual review and the natural language processing pipeline in the test data set (N=1000).

Extracted indicators			Human annotator											Method							*P* value^a^
			A		B		C		D		E		Manual review^b^			NLP system		Gold standard^c^			
**Detection rate, n (%)**																					
	ADR^d^	467 (46.7)		474 (47.4)		474 (47.4)		475 (47.5)		468 (46.8)		472 (47.2)			468 (46.8)		475 (47.5)		.92		
	SDR^e^	65 (6.5)		64 (6.4)		66 (6.6)		64 (6.4)		64 (6.4)		65 (6.5)			64 (6.4)		66 (6.6)		.99		
**Surveillance interval group, n (%)**																					
	1 year	N/A^f^		N/A		N/A		N/A		N/A		N/A			N/A		N/A		N/A		
	3 years	59 (93.7)		58 (92.1)		60 (95.2)		62 (98.4)		58 (92.1)		59 (93.6)			63 (100)		63 (100)		.92		
	3-5 years	62 (89.9)		67 (97.1)		64 (92.8)		63 (91.3)		68 (98.6)		65 (93.9)			68 (94.2)		69 (100)		.92		
	5-10 years	40 (100)		40 (100)		40 (100)		40 (100)		40 (100)		40 (100)			39 (97.5)		40 (100)		.99		
	7-10 years	339 (97.7)		347 (100)		345 (99.4)		345 (99.4)		346 (99.7)		344 (99.1)			343 (98.9)		347 (100)		.99		
	10 years	479 (99.6)		480 (99.8)		481 (100)		480 (99.8)		480 (99.8)		480 (99.8)			480 (99.8)		481 (100)		.99		

^a^*P* values were calculated using the 2X3 chi-square test.

^b^Mean of the judgments made by the 5 human annotators.

^c^Consensus judgment of the 5 human annotators; applied in inconsistent cases.

^d^ADR: adenoma detection rate.

^e^SDR: sessile serrated lesion detection rate.

^f^N/A: not applicable (no patients were assigned a 1-year surveillance interval).

### Analysis of ADR, SDR, and Surveillance Intervals in a 10-Year Colonoscopy Report Data Set

The NLP pipeline was applied to a set of 54,562 colonoscopy reports (and their associated pathology reports) created by 25 endoscopists who examined patients aged ≥50 years over a 10-year period; the NLP analyzed ADR, SDR, and surveillance intervals in the reports (Table 4). The overall ADR, advanced ADR, SDR, and advanced SDR were 42% (22,909/54,562), 3.4% (1838/54,562), 3.3% (1806/54,562), and 0.5% (248/54,562), respectively. The difference in detection rate between the endoscopists with the highest and lowest performance was 39.9% (1055/1876, 56.2% vs 264/1615, 16.3%, respectively) for ADR, 5.3% (83/1165, 7.1% vs 30/1615, 1.8%, respectively) for advanced ADR, 6.2% (124/1876, 6.6% vs 6/1615, 0.4%, respectively) for SDR, and 1.6% (11/679, 1.6% vs 0/1615, 0%, respectively) for advanced SDR. Overall, the mean surveillance interval was 8.7 years, and the difference in the surveillance interval assigned by endoscopists with the highest and lowest performance was 1.3 years (9.5 years vs 8.2 years). Table 5 shows the proportion of patients assigned to each of the 6 surveillance interval groups by groups of endoscopists divided according to the endoscopists’ ADR and SDR. The group of endoscopists with the lowest ADR (<30%) assigned a higher proportion of patients to the longest surveillance interval than did the endoscopists with the highest ADR (>45%). This pattern was similar for the endoscopists with the highest and lowest SDR.

**Table 4:** Clinical application of the natural language processing pipeline to nonannotated colonoscopy data created by 25 endoscopists between 2010 and 2019.

Endoscopist	Procedures	Adenoma detection rate, n (%)	Advanced adenoma detection rate, n (%)	Sessile serrated lesion detection rate, n (%)	Advanced sessile serrated lesion detection rate, n (%)	Mean surveillance interval, years
A	3060	1112 (36.3)	94 (3.1)	58 (1.9)	8 (0.3)	8.9
B	981	343 (35)	36 (3.7)	8 (0.8)	0 (0)	9.0
C	3553	1447 (40.7)	129 (3.6)	91 (2.6)	21 (0.6)	8.8
D	2765	1109 (40.1)	92 (3.3)	83 (3)	17 (0.6)	8.8
E	1174	469 (39.9)	46 (3.9)	18 (1.5)	3 (0.3)	8.9
F	1258	338 (26.9)	39 (3.1)	21 (1.7)	1 (0.1)	9.2
G	679	301 (44.3)	12 (1.8)	40 (5.9)	11 (1.6)	8.6
H	1165	505 (43.3)	83 (7.1)	21 (1.8)	4 (0.3)	8.4
I	1615	264 (16.3)	30 (1.9)	6 (0.4)	0 (0)	9.5
J	2091	917 (43.9)	43 (2.1)	92 (4.4)	12 (0.6)	8.7
K	1876	1055 (56.2)	58 (3.1)	124 (6.6)	16 (0.9)	8.2
L	3284	1739 (53)	73 (2.2)	144 (4.4)	14 (0.4)	8.4
M	3437	1510 (43.9)	116 (3.4)	132 (3.8)	3 (0.1)	8.6
N	3799	1708 (45)	119 (3.1)	130 (3.4)	13 (0.3)	8.6
O	647	292 (45.1)	14 (2.2)	14 (2.2)	1 (0.2)	8.8
P	1707	844 (49.4)	74 (4.3)	87 (5.1)	16 (0.9)	8.4
Q	2964	1435 (48.4)	106 (3.6)	137 (4.6)	16 (0.5)	8.5
R	3209	1235 (38.5)	108 (3.4)	99 (3.1)	12 (0.4)	8.8
S	2168	816 (37.6)	52 (2.4)	61 (2.8)	8 (0.4)	8.9
T	3834	1633 (42.6)	119 (3.1)	152 (4)	23 (0.6)	8.7
U	3935	1324 (33.6)	127 (3.2)	68 (1.7)	9 (0.2)	9.1
V	1936	1014 (52.4)	114 (5.9)	104 (5.4)	17 (0.9)	8.2
W	643	268 (41.7)	33 (5.1)	4 (0.6)	0 (0)	8.8
X	1469	680 (46.3)	65 (4.4)	73 (5)	16 (1.1)	8.5
Y	1313	551 (42)	56 (4.3)	39 (3)	7 (0.5)	8.7
Total	54,562	22,909 (42)	1838 (3.4)	1806 (3.3)	248 (0.5)	8.7

**Table 5:** Proportion of patients assigned different surveillance intervals, sorted by endoscopists (N=25) with high, medium, and low adenoma detection rates and sessile serrated lesion detection rates.

Surveillance interval	Adenoma detection rate, n (%)				Sessile serrated lesion detection rate, n (%)		
	<30% (n=2873)	30%-45% (n=37,806)	>45% (n=13,883)	<2% (n=13,831)		2%-4% (n=24,725)	>4% (n=16,006)
1 year	0 (0)	14 (0.04)	13 (0.09)	3 (0.02)		8 (0.03)	16 (0.1)
3 years	77 (2.68)	1918 (5.07)	894 (6.44)	603 (4.36)		1284 (5.19)	1002 (6.26)
3-5 years	59 (2.05)	2204 (5.83)	1217 (8.77)	545 (3.94)		1557 (6.3)	1378 (8.61)
5-10 years	25 (0.87)	670 (1.77)	389 (2.80)	138 (1.00)		491 (1.99)	455 (2.84)
7-10 years	472 (16.43)	11,213 (29.66)	4953 (35.68)	3527 (25.5)		7508 (30.37)	5603 (35.01)
10 years	2231 (77.75)	21,740 (57.5)	6397 (46.08)	8988 (64.98)		13,851 (56.02)	7529 (47.04)

## Discussion

### Comparison With Other NLP Systems

There have been various efforts to develop NLP systems for monitoring the quality of colonoscopies in Western countries, and these have shown excellent performance in measuring procedure indications, cecal intubation rate, and the presence and location of polyps. NLP systems have been studied that have various levels of complexity and perform various tasks, ranging from simple extraction tasks, such as assessing the presence and location of polyps, to the automated extraction and calculation of quality metrics [23-31]. However, Western-developed NLP systems in previous studies were based on reports written in English and used NLP lexicons from common language systems, such as the unified medical language system and the Systematized Nomenclature of Medicine-Clinical Terms. These systems cannot be applied to a set of reports written in Korean, both Korean and English, and English only, such as the one examined in this study. Therefore, for the first time in Korea, we developed an NLP pipeline to process colonoscopy reports written in multiple languages. A lexicon including Korean and English medical terms and various endoscopic abbreviations was used to construct the NLP pipeline. Hence, our NLP pipeline processed reports with feasible performance in the validation data set for capturing key quality indicators, including the detection rate for SSLs (previous NLP systems have only captured a few SSLs).

We demonstrated the clinical application of the NLP pipeline with a 10-year set of nonannotated colonoscopy reports. Quality indicators, including ADR, SDR, and surveillance intervals, were extracted from reports written by 25 gastroenterologists, and the proportion of patients assigned different surveillance intervals was analyzed to determine the quality of polyp detection by the endoscopists. We found that ADR and SDR had great variance among the endoscopists, a result that is in line with previous studies [2-4]. There was a 3.4-fold variation in ADR between the endoscopists with the lowest and highest levels (1055/1876, 56.2% vs 264/1615, 16.3%, respectively) and a 16.5-fold variation in SDR (124/1876, 6.6% vs 30/1615, 0.4%, respectively).

### Importance of SSL Detection and Performance Feedback

Although awareness of the clinical importance of SSLs for colorectal cancer via the serrated pathway has increased since 2010, our data revealed that detecting SSLs remains a challenge for endoscopists performing screening colonoscopies. SSLs typically show a subtle endoscopic appearance: they can be flat, mucus-coated, and have indistinct borders, which is a totally different appearance from conventional adenomas [32]. Most recently, Lee et al [3] reported the results of a 1-year educational intervention based on a computerized training module that imparted knowledge on the appearance of SSLs using the NICE (Narrow Band Imaging International Colorectal Endoscopic) and WASP (Workgroup on Serrated Polyps and Polyposis) classifications. In this large study, which included 15 experienced endoscopists, the SDR improved significantly, from 4.5% at baseline to 7.1%. Therefore, implementing an NLP system for colonoscopies in clinical practice could provide feedback on the detection performance of individual endoscopists in real time and motivate endoscopists to improve their knowledge and observation techniques for difficult polyps.

### Optimization of Surveillance Interval Recommendations

Current surveillance interval recommendations for follow-up colonoscopies do not consider the performance of the physician and only consider the characteristics of the removed polyp. Our study reveals that the recommended surveillance interval can be incorrectly long, depending on the performance level of the endoscopist. High-performance endoscopists (ADR >45%) recommended a 10-year surveillance interval in 46.1% of patients (6397/13,883), while low-performance endoscopists (ADR <30%) recommended a 10-year surveillance interval in 77.8% of patients (2231/2873). This wide difference in the proportion of patients that received a recommendation of a 10-year surveillance interval suggests that low-performance endoscopists missed polyps, negatively affecting their calculation of the future risk of patients and leading them to recommend an inappropriately long surveillance interval. Therefore, endoscopists should periodically check their own ability to detect neoplastic polyps and adjust their recommendations for surveillance interval according to their level of performance to prevent cancer development. Colonoscopy NLP systems could have a role in this self-evaluation process, providing an essential clinical decision support system and enabling the optimal choice of surveillance intervals by considering not only the risk of the patient, but also the performance of the endoscopist.

### Limitations

This study has the following limitations: First, it was conducted at a single center, leaving open the possibility that the NLP pipeline may not be able to properly process colonoscopy reports retrieved from other centers. As the NLP pipeline is based on regular expression rules formulated from linguistic patterns in the development data set, terms or patterns in other reports that are not present in the development data set can result in false processing of the reports. Second, the integrity of the NLP pipeline depends on the endoscopist’s documentation practice. For example, miswriting orders, numbers, or the count of the biopsied polyps could create mismatches between a colonoscopy report and its associated pathology report, resulting in false processing in the pipeline. However, this is not a problem unique to our study; it applies to all projects that use current NLP pipelines. Therefore, future research may be required to develop more confident NLP systems that warn of the possibility of false processing or to develop more sophisticated systems based on deep learning approaches and cutting-edge NLP models, such as bidirectional encoder representations from transformers (BERT) [33].

### Conclusions

In summary, we developed an NLP pipeline to transform multi-language, free-text reports into a structured format to automate the calculation of quality indicators. The NLP pipeline processed the validation data set with high performance that was similar to a manual review performed by experts. The NLP-derived information from a 10-year real-world data set found that individual endoscopists showed great variance in quality indicators and patient risk stratification. This automated NLP process could be a useful decision support system for endoscopists, as it could allow the optimal recommendation of postcolonoscopy surveillance intervals based on both patient risk and endoscopist performance. This system could positively impact the quality of colonoscopy in many hospitals and health check-up centers that conduct screening programs. Furthermore, information extracted by NLP pipelines from big data derived from colonoscopy reports should be a valuable resource for research into the association of colon polyps with various diseases and into guideline adherence patterns.

## Abbreviations

ADR: adenoma detection rate
NLP: natural language processing
SDR: sessile serrated lesion detection rate
SSL: sessile serrated lesion
